# Berlyne Revisited: Evidence for the Multifaceted Nature of Hedonic Tone in the Appreciation of Paintings and Music

**DOI:** 10.3389/fnhum.2016.00536

**Published:** 2016-11-04

**Authors:** Manuela M. Marin, Allegra Lampatz, Michaela Wandl, Helmut Leder

**Affiliations:** ^1^Department of Basic Psychological Research and Research Methods, University of ViennaVienna, Austria; ^2^Department of Psychology, University of InnsbruckInnsbruck, Austria

**Keywords:** esthetic experience, neuroaesthetics, beauty, pleasantness, liking, complexity, visual arts

## Abstract

In his seminal book on esthetics, Berlyne (1971) posited an inverted-U relationship between complexity and hedonic tone in arts appreciation, however, converging evidence for his theory is still missing. The disregard of the multidimensionality of complexity may explain some of the divergent results. Here, we argue that definitions of hedonic tone are manifold and systematically examined whether the nature of the relationship between complexity and hedonic tone is determined by the specific measure of hedonic tone. In Experiment 1, we studied three picture categories with similar affective and semantic contents: 96 affective environmental scenes, which were also converted into 96 cartoons, and 96 representational paintings. Complexity varied along the dimension of elements. In a between-subjects design, each stimulus was presented for 5 s to 206 female participants. Subjective ratings of hedonic tone (either beauty, pleasantness or liking), arousal, complexity and familiarity were collected in three conditions per stimulus set. Complexity and arousal were positively associated in all conditions, with the strongest association observed for paintings. For environmental scenes and cartoons, there was no significant association between complexity and hedonic tone, and the three measures of hedonic tone were highly correlated (all *r*_s_ > 0.85). As predicted, in paintings the measures of hedonic tone were less strongly correlated (all *r*_s_ > 0.73), and when controlling for familiarity, the association with complexity was significantly positive for beauty (*r*_s_ = 0.26), weakly negative for pleasantness (*r*_s_ = -0.16) and not present for liking. Experiment 2 followed a similar approach and 77 female participants, all non-musicians, rated 92 musical excerpts (15 s) in three conditions of hedonic tone (either beauty, pleasantness or liking). Results indicated a strong relationship between complexity and arousal (all *r*_s_ > 0.85). When controlling for familiarity effects, the relationship between complexity and beauty followed an inverted-U curve, whereas the relationship between complexity and pleasantness was negative (*r*_s_ = -0.26) and the one between complexity and liking positive (*r*_s_ = 0.29). We relate our results to Berlyne’s theory and the latest findings in neuroaesthetics, proposing that future studies need to acknowledge the multifaceted nature of hedonic tone in esthetic experiences of artforms.

## Introduction

Esthetic experiences are phenomena that are inherently challenging to study due to a myriad of underlying factors. Therefore, it is not surprising that the field of empirical esthetics has generated competing accounts for why humans show preferences for certain objects over others or why they consider some objects as more beautiful (Palmer et al., 2013; Pelowski et al., 2016). For example, the mere exposure effect (Zajonc, 1968) suggests that the higher the frequency of exposure, the more the stimulus is liked, whereas Berlyne’s psychobiological model (Berlyne, 1971) emphasizes the mediating role of arousal in the relationship between stimulus properties and measures of hedonic value (e.g., esthetic pleasure or preference) or interest. Alternatively, Martindale and Moore (1988) and Martindale et al. (1990) propose that more prototypical exemplars of a category are preferred to non-prototypical ones. Fluency theory (Reber et al., 2004; Reber, 2012), which posits that the ease of processing determines preference, may also explain the underlying mechanism of the mere exposure effect and the prototype-preference theory, but it does not offer a clear account for Berlyne’s arousal theory (Palmer et al., 2013). Lately, the field has witnessed the emergence of several multicomponent models (Leder et al., 2004; Hagtvedt et al., 2008; Marković, 2012; Shimamura, 2012; Tinio, 2013; Chatterjee and Vartanian, 2014; Leder and Nadal, 2014; Redies, 2015; Pelowski et al., 2016), which aim to describe the essence of esthetic experiences by modeling the relationship between bottom–up and top–down processes as well as their underlying causes and neural substrates. Here, we follow this approach with a strong emphasis on Berlyne’s theory.

The field of empirical esthetics has generated a growing body of literature, including divergent results. To be sure, Berlyne’s (1971, 1974a) psychobiological model of esthetic experience and his *New Experimental Esthetics* have been very influential in the field (Konečni, 1978; Silvia, 2005), but besides empirical support for his theory a non-negligible number of conflicting results have also been reported (Vitz, 1964; Osborne and Farley, 1970; Kreitler et al., 1974; Nicki and Gale, 1977; Krupinski and Locher, 1988; Neperud and Marschalek, 1988; Messinger, 1998; Stamps Iii, 2002; Eisenberg and Thompson, 2003; Nadal et al., 2010; Marin and Leder, 2013). Therefore, the current research project sought to contribute to the emerging literature by offering explanations for the discrepant research findings regarding Berlyne’s theory. For instance, contributions of the multidimensionality of visual complexity (Nadal et al., 2010), individual differences in esthetic preferences (Güçlütürk et al., 2016) as well as a dual-process perspective on preference formation (Graf and Landwehr, 2015) have recently been acknowledged. In this study, we aim to explore alternative pathways in the elucidation of Berlyne’s psychobiological model: First, by following a stringent comparative approach, we study the nature of hedonic tone and its relationship with complexity in the experience of three different sets of affective pictures varying in their esthetic quality. Second, we also study the nature of hedonic tone in the appreciation of music using a similar approach. Third, we link our findings to current research in the field of neuroaesthetics.

Being interested in curiosity and exploratory behavior, Berlyne follows an information-theoretic approach and regards the structural features of an artwork as a primary source of esthetic response. To be specific, his psychobiological model is based on Wundt’s (1874) curve and suggests that a set of psychophysical, ecological, and collative stimulus features have a certain arousal potential, which in turn determines hedonic value. Very low and high arousal potential leads to low levels of hedonic value, whereas moderate levels lead to high hedonic value, yielding an inverted-U curve which is generated by interacting arousal systems in the brain. According to Berlyne, collative variables, such as novelty, complexity, uncertainty and conflict, are those with the highest arousal potential in the perceiver. The term “hedonic value” comprises several distinct variables, such as pleasure (pleasantness), preference, utility, which can be measured by verbal ratings, as well as reward value and incentive value, which can be measured by non-verbal behavior. A related attribute is beauty (Berlyne, 1971), whose relationship with collative variables has also been frequently studied (Nadal et al., 2010). Berlyne (1974a) later introduced the term “hedonic tone” to refer to verbal expressions of pleasure and the like. Importantly, Berlyne (1974a) states that these measures of hedonic value may or may not have the same underlying psychophysiological basis. Therefore, our findings will have important implications for a better understanding of the neural underpinnings of esthetic experiences (Pearce et al., 2016).

Berlyne’s theory has been put to test with a wide range of stimuli, ranging from abstract patterns to paintings and from simple melodic sequences to real music (for a review see (Nadal, 2007; Nadal et al., 2010; Marin and Leder, 2013). Although Berlyne’s psychobiological model has been fruitful in the study of psychology and the arts (Silvia, 2005; Jacobsen, 2006), it has also generated criticism, mostly stemming from the fact that the inverted-U shape relationship between collative variables and hedonic value could not always be replicated. For example, Cupchik (1988) regretted that Berlyne’s skepticism about cognitive psychology hindered the study of how thought and emotion interact during esthetic experiences, which implies a disinterest in the study of understanding, a critical attribute of art perception (Leder et al., 2004). Konečni (1996) criticized Berlyne for not paying enough attention to sympathetic arousal and emotion as well as to the context of esthetic behavior. Similarly, Silvia (2005) pointed out that an arousal theory cannot account for the diversity of emotions experienced during art perception. Furthermore, the concept of arousal that Berlyne had in mind has not been supported by neurophysiological research (Nicki, 1972; Nicki and Gale, 1977; Silvia, 2005; Nadal, 2007), and others proposed that prototypicality is better able to explain esthetic preference than a collative motivation model (Whitfield, 1983; Martindale and Moore, 1988). Last but not least, it has been suggested that single-factor explanations, in this case the modulation of arousal through a set of stimulus features, may not be sufficient to capture the nature of esthetic experiences (Leder and Nadal, 2014).

However, these objections do not justify abandoning Berlyne’s theory, especially since there is cumulative evidence that arousal plays an important role, not only in emotions in general (Russell, 1980), but specifically in esthetic experiences. First, it should be pointed out that discrepancies regarding Berlyne’s theory seem mostly relate to the inverted-U shape relationship between collative variables and hedonic value, and not to the predicted linear relationship between collative variables and subjective arousal. Even in studies that could not confirm the inverted-U shape relationship, a positive relationship between collative variables and subjective arousal was reported when artistic (Krupinski and Locher, 1988; Neperud and Marschalek, 1988; Marin and Leder, 2013) and non-artistic stimuli (Heath et al., 2000; Marin and Leder, 2013) were investigated. This suggests that a differentiated view on Berlyne’s theory is required. Second, arousal has recently been identified as a mediator between the relationship of complexity and pleasantness for visual stimuli, and to a lesser degree, for music (Marin and Leder, 2013, 2016). Third, there is a large body of empirical evidence, not only in light of Berlyne’s model, suggesting a relationship between arousal and stimulus features in the visual as well as auditory domain (Jacobs and Hustmyer, 1974; Bertamini et al., 2013; Marin and Leder, 2013; Gingras et al., 2014). Fourth, a link between arousal and preference has frequently been reported (Konečni et al., 1976; Konečni and Sargent-Pollock, 1977; North and Hargreaves, 2000; Schäfer and Sedlmeier, 2011; Blijlevens et al., 2012; Ramsøy et al., 2012). Bearing in mind that arousal may generally affect judgment, learning and memory (Storbeck and Clore, 2008), we thus propose that the concept of arousal should be more systematically studied in the context of Berlyne’s theory, especially since alternative theories, such as appraisal theory (Silvia, 2005), also include an arousal component.

Research in the line of Berlyne (1974a) has originally followed a program using verbal ratings as well as psychophysiological and behavioral measures as dependent variables. Verbal ratings usually comprised a large set of *descriptive scales* (measuring collative variables), *evaluative scales* (measuring hedonic value and related attributes), *internal-state scales* (measuring arousal, tension and pleasure) as well as, to a lesser degree, *stylistic scales* (referring to stylistic features of an artwork). Factor analysis conducted on these variables often showed pleasure to be correlated with other evaluative scales (see Berlyne, 1974a). Early research in the tradition of Berlyne’s *New Experimental Esthetics* thus usually worked with more than one rating scale for each scale category, which has become less usual in recent times. Nowadays, a small set of scales and items is generally used to study esthetic experiences in the context of Berlyne’s theory, probably due to the fact that research has shown that Berlyne’s large set of scales can be reduced to 2–3 factors (e.g., hedonic tone, arousal, and complexity) (Berlyne, 1974a). Although a reduction in the number of rating scales is generally welcome, especially because it allows for simpler research designs and shorter experiments, many researchers after Berlyne do not explain why they have chosen one rating scale out of several possible ones. In other words, it is often unclear why authors chose to measure beauty instead of liking or pleasantness as a measure of hedonic tone. Therefore, it can be argued that the multifaceted nature of hedonic tone (by which we refer to the many aspects this term originally comprised) has not received enough attention in the field. We believe that it is necessary to acknowledge the concepts behind different measures of hedonic tone and to study their relationships with complexity in different stimulus sets varying in esthetic quality.

The main goal of the current study was to clarify how verbal reports of different measures of hedonic tone relate with visual and musical complexity, aiming to contribute to the elucidation of Berlyne’s theory (Nadal et al., 2010; Marin and Leder, 2013; Güçlütürk et al., 2016). Recent empirical work has demonstrated that the relationship between complexity and beauty can be explicated by considering the multidimensionality of complexity (Nadal et al., 2010), which is the finding that the experience of complexity can be produced by variations of (1) the number and diversity of elements, (2) disorganization and (3) asymmetry. Nadal et al. (2010) showed that if complexity is varied along the dimension of elements, a positive linear relationship with beauty is observed. Variation along the dimension of disorganization revealed a U-shape relationship with beauty, whereas indications for an inverted-U shape relationship were found for variation along the dimension of symmetry. We were inspired by this approach and explored the possibility that the specific measure of hedonic tone may determine the relationship with a specific dimension of complexity.

Concepts such as complexity, arousal, pleasantness, liking, and beauty are also essential features of more recent models of esthetic experiences (Leder et al., 2004; Brattico et al., 2013; Leder and Nadal, 2014; Pelowski et al., 2016), thus the study of their interrelationships is of general interest to the field. If it can be shown that complexity and measures of hedonic tone are associated in different ways, and importantly, that measures of hedonic tone are related, the data would directly justify the integration of these concepts into one model of esthetic experience. Importantly, the outcome of the current study may have direct consequences for linking cognitive models of esthetic experience with current neuroimaging data (Cela-Conde et al., 2013; Chatterjee and Vartanian, 2014; Leder et al., 2015). In the last decade, neuroimaging studies on the appreciation of visual art have suggested that, rather than a single brain area, there is a complex neural system that underlies esthetic experiences (Nadal, 2013; Cela-Conde and Ayala, 2014; Vartanian and Skov, 2014; Boccia et al., 2016).

Nadal (2013) suggested that at least three different sets of brain regions underlie esthetic experiences, and these brain regions may be differently involved in the processing of hedonic tone. Evaluative judgments, attentional processing and memory retrieval are related to activations in the prefrontal, parietal and temporal cortical regions. Esthetic experiences also involve the reward circuit as well as low-, mid-, and high-level cortical sensory regions (see also Cela-Conde et al., 2013). In the context of the current study, brain regions related to evaluative judgments and the reward circuit are of particular interest. Activation of the dorsolateral prefrontal cortex (Cela-Conde et al., 2004) and the anterior medial prefrontal cortex (Jacobsen and Höfel, 2003; Jacobsen et al., 2006; Kirk, 2008) may indicate the engagement of evaluative judgment processes, in this case mostly related to beauty. Nadal (2013) suggested that, since these activations occur between 400 and 600 ms after picture onset, they may be involved in the formation of initial impressions which impact on further processing stages such as attention, perception and response selection (Cela-Conde et al., 2011). Other brain areas reported to be active during evaluative judgments (of beauty or appeal) are the ventrolateral prefrontal cortex (Jacobsen et al., 2006; Kirk, 2008), the left temporal pole (Jacobsen et al., 2006), as well as the posterior cingulate cortex and precuneus (Jacobsen et al., 2006; Kirk et al., 2009).

Several neuroimaging reports on the esthetic experience of art have also confirmed activation of different brain areas constituting the reward circuit (Kawabata and Zeki, 2004; Kirk, 2008; Ishizu and Zeki, 2011; Lacey et al., 2011), which may be related to the experience of pleasure or pleasantness as well as liking responses. The activity of the medial orbitofrontal cortex was positively associated with liking or preference (Kawabata and Zeki, 2004; Kirk, 2008), whereas the activity in the lateral orbitofrontal cortex was negatively associated with appeal ratings (Kirk, 2008; Munar et al., 2012). Another brain area of the reward circuit, the anterior cingulate cortex, was found to be active when people were engaged with an artwork they liked (Vartanian and Goel, 2004; Cupchik et al., 2009; Kirk et al., 2009; Boccia et al., 2015; Yeh et al., 2015). Both the orbitofrontal cortex and the anterior cingulate cortex were active when cognitive and affective processes interacted with each other during the evaluation of sensory information (Pessoa, 2008; Rolls and Grabenhorst, 2008). The insular cortex was more active when viewers experienced emotions induced by an artwork. Moreover, sub-cortical components of the reward circuit, such as the nucleus accumbens, have frequently been reported to be active during pleasurable esthetic experiences (Vartanian and Goel, 2004; Cupchik et al., 2009; Kirk et al., 2009; Ishizu and Zeki, 2011). Taken together, these neuroimaging studies suggest that judgments of beauty, pleasantness and liking may not be necessarily processed by the same set of brain regions. Similar conclusions have also been drawn by Brattico et al. (2013) who modeled the neural correlates of music-induced esthetic experiences.

There is a recent interest in comparing esthetic experiences across object classes and sensory domains (for a review see Marin, 2015), and Berlyne’s psychobiological model, regardless of the debate about its underlying mechanisms, may be adopted as a scientific playground to study the nature of esthetic experiences (Normore, 1974; Marin and Leder, 2013). Berlyne’s theory made clear predictions and encompasses general concepts that can meaningfully be studied in different sensory domains and in different object classes within one sensory domain. Recently, by studying affective environmental scenes, representational paintings and music, we found that the relationship between complexity and arousal seems to be stronger in the musical than in the visual domain (Marin and Leder, 2013). We showed that mediation effects of arousal may be more prominent in the visual than in the musical domain, and further, that objective measures of visual complexity can be used in their analogous forms for studying musical complexity. Regarding the relationship between hedonic tone (pleasantness) and complexity, a complex interplay between familiarity, participant gender and stimulus type determined the kind of relationship. Neither the predictions made by Berlyne (1971) (inverted-U shape) nor those of Nadal et al. (2010) (linear positive for variations of the number of elements) were confirmed. Since pleasantness, the measure of hedonic tone, was also decisive in the pre-selection of the affective stimuli, we argued that other measures of hedonic tone should be used to study the relationship with complexity (Marin and Leder, 2013). In other words, not only the multidimensional nature of complexity may play a role in the relationship with hedonic tone but also the type of hedonic measure (e.g., pleasantness, liking, beauty etc.).

The results of previous studies using more than one measure of hedonic tone in the visual domain indeed suggest that the interrelationships between them may vary according to the stimulus type. For example, Berlyne and Ogilvie (1974) asked participants to rate a set of paintings stemming from the 14th to the 20th century on 12 ratings scales, including beauty, pleasantness, and pleasure. Importantly, they found that these scales, although loading on the same hedonic tone factor, correlated somewhat differently with the complexity scale in their two small groups of participants, making it difficult to draw any conclusions. Normore (1974) studied animations of a dot whose dynamics regarding position, brightness and duration varied in complexity. In comparison to Berlyne and Ogilvie (1974) study, ratings of beauty and pleasingness were nearly identical. Berlyne (1974b) also investigated visual patterns and, among other things, made participants rate beauty, pleasingness and liking. He observed that beauty and pleasingness were highly positively correlated, which also held true for the relationship between beauty and liking as well as for the relationship between pleasingness and liking. In a between-subjects design, Russell and George (1990) further investigated evaluative responses to 15 paintings of diverse Western styles by obtaining ratings of preferability, pleasingness, likability, complexity, and familiarity. The negative correlation between complexity and pleasingness was stronger than those correlations observed for preferability and likability. Likability and preferability as well as likability and pleasingness were highly correlated, whereas the relationship between pleasingness and preferability was slightly weaker, but these correlations were not corrected for familiarity effects. Looking at architectural stimuli, Imamoglu (2000) investigated esthetic responses to different types of house façade drawings and collected ratings of pleasantness, beauty and liking. The results indicated that the three measures of hedonic tone were highly correlated. Sato and Oda (2014) studied the effects of shape and color on the esthetic evaluation of colored shapes varying in complexity and curvature. The results showed that shape and color affected beauty, pleasantness and liking ratings slightly differently. In summary, these findings suggest that the interrelationship between the three measures of hedonic tone may be stronger for naturalistic pictures and abstract patterns than for artworks.

In the current study, we followed a systematic comparative approach – across stimulus categories – and sought to examine (i) the nature of hedonic tone and its relationship with visual complexity as defined by the number and diversity of elements, and (ii) the nature of hedonic tone and its relationship with musical complexity. We studied three evaluative scales frequently used as measures of hedonic tone, namely pleasantness, beauty and liking. In Experiment 1, we collected subjective reports on affective environmental scenes (IAPS pictures) and representational paintings (Marin and Leder, 2013), in which complexity was mostly varied along the dimension of elements (Marin and Leder, 2016). Environmental scenes were photographs of everyday-life scenes, involving a range of semantic categories and various emotional contents, which are widely used to study emotion and attention. Representational paintings, mostly stemming from the 19th century, also comprised a wide range of semantic and emotional contents but were not matched on a one-to-one basis with environmental scenes. Although these two picture sets were similar in semantic and emotional contents, and thus allowed for some conclusions about the nature of esthetic experiences, we also introduced a new, third picture set, namely environmental scenes transformed into cartoons by means of photo editing software. These cartoon-like pictures thus contain identical semantic content, preserve composition and ordering of objects, but also render a visually poorer, reduced representation, and lack the distinctive visual features that mark an artist’s style. This enabled us to compare two picture sets that differed in their artistic quality but not necessarily in their emotional and semantic contents. Moreover, the cartoons represented a uniform “artistic” style, which was not exactly the case for representational paintings. In Experiment 2, we used musical stimuli taken from Experiment 3 as described in Marin and Leder (2013) and followed a similar procedure as in Experiment 1.

We decided to employ a between-subjects design in which participants rated pictures or musical excerpts for familiarity, complexity, and arousal as well as for only one of the three measures of hedonic tone. This ensured that participants could not guess the aim of the experiment, and further, this design was also closer to what other researchers mostly employed in their designs, i.e., to use only one measure of hedonic tone. Last, we decided to restrict our sample to females because gender effects regarding these picture sets have been reported earlier (Marin and Leder, 2013). We also controlled for mood prior to the experiment (Flexas et al., 2013; Gartus and Leder, 2014) as well as for interest in visual arts (Experiment 1) and musical sophistication (Experiment 2).

For Experiment 1 we hypothesized that, in line with Nadal et al. (2010), beauty would show a positive association with complexity as defined by the number and diversity of elements. Based on Marin and Leder (2013, 2016), we predicted that there would be no significant relationship between this complexity dimension and pleasantness in environmental scenes and paintings if familiarity is controlled for. Regarding liking ratings and the relationship with complexity it was difficult to make a concrete prediction, except for arguing for an inverted-U shape relationship, following Berlyne’s theory. In addition, we surmised that the relationship between complexity and arousal would be positive in all picture sets but stronger in paintings than in environmental scenes and cartoons (Marin and Leder, 2013). Furthermore, we hypothesized that the multifaceted nature of hedonic tone in relation to complexity may be most obvious regarding visual arts in comparison to environmental scenes and cartoons (Berlyne and Ogilvie, 1974; Russell and George, 1990; Imamoglu, 2000).

For Experiment 2, our hypotheses were less concrete because much less is known about the underlying dimensions of musical complexity. However, as objective measures of musical complexity have indicated in Marin and Leder (2013), event density moderately correlated with ratings of subjective complexity. Based on this finding and findings by Nadal et al. (2010), we predicted a positive association between beauty and complexity, assuming that complexity and beauty are similarly related in both the visual and musical domains. Following findings by Marin and Leder (2013) for the group of females, we predicted no significant association between pleasantness and complexity after controlling for familiarity effects. For the complexity-liking association we were only able to refer to Berlyne’s original theory and his predicted inverted U-shape relationship. Furthermore, we predicted a strong relationship between complexity and arousal in all experimental conditions.

## Experiment 1

### Materials and Methods

#### Participants

Two-hundred thirty female psychology students at the University of Vienna participated in the experiment in exchange for course credit. All participants were unfamiliar with the three picture sets and did not participate in previous studies by Marin and Leder (2013, 2016). Participants had normal or corrected-to-normal visual acuity and normal color vision. The participants were randomly assigned to one of nine groups that differed regarding the three picture sets and the type of hedonic measure to be rated (beauty, pleasantness and liking).

The participants of the nine groups were screened for outliers with respect to their mood prior to the experiment (participants with very low mood scores were removed), their art interest as well as the trait emotional intelligence, empathy and stress reactivity scales (SRS; several participants scored very low on the empathy and emotional intelligence scales). Twenty-four participants were removed for these reasons by investigating the respective boxplots (an outlier was defined as a data point more than 1.5 interquartile ranges below the first quartile or above the third quartile). The remaining 206 participants in the nine groups did not significantly differ regarding their age, mood prior to the experiment and their scores on the trait EI, empathy quotient and SRS. We also ensured that art interest was similar, and generally low, in the six groups rating cartoons and paintings (see **Table 1**).

**Table 1:** Participant characteristics.

Group	Age [years]		Pos./neg. mood A	Alertness/fatigue A	Quietude/disquietude A	Art interest	Trait EI	EQ short	SRS
IAPS Beauty	*M*	23.8	17.29	13.00	15.08	–	156.88	70.63	58.13
(*n* = 24)	*SD*	4.0	1.81	2.95	3.31		16.39	7.30	8.72

IAPS Pleasantness	*M*	22.1	17.57	13.14	16.43	–	163.52	71.67	57.00
(*n* = 21)	*SD*	2.9	2.18	3.81	2.62		10.41	5.03	8.46

IAPS Liking	*M*	22.2	16.22	12.65	15.26	–	151.17	70.61	59.70
(*n* = 23)	*SD*	3.3	3.01	2.99	2.49		13.23	5.65	9.76

Cartoons Beauty	*M*	24.3	17.04	14.44	14.96	63.40	153.48	71.04	56.96
(*n* = 25)	*SD*	5.3	2.54	3.83	3.18	16.64	18.12	6.41	8.17

Cartoons Pleasantness	*M*	21.8	16.64	12.73	14.82	64.05	157.50	72.95	59.72
(*n* = 22)	*SD*	1.9	2.50	3.40	2.46	11.94	12.69	5.07	9.32

Cartoons Liking	*M*	22.6	17.29	14.04	16.13	59.38	160.46	72.42	57.38
(*n* = 24)	*SD*	2.4	1.94	3.30	2.76	15.10	13.70	5.91	8.84

Paintings Beauty	*M*	22.6	16.41	12.77	15.18	62.00	158.45	73.14	61.73
(*n* = 22)	*SD*	4.0	2.58	3.57	3.06	14.40	15.85	6.29	7.75

Paintings Pleasantness	*M*	22.4	17.35	13.30	15.40	66.95	154.65	70.90	60.95
(*n* = 20)	*SD*	4.0	1.84	2.66	2.54	11.17	14.38	5.92	8.13

Paintings Liking	*M*	22.2	16.92	13.64	14.84	61.24	153.04	69.64	57.96
(*n* = 25)	*SD*	3.1	1.89	4.28	2.51	15.03	22.77	7.30	8.87

Test statistic	*H* = 10.49		*H* = 5.55	*H* = 6.59	*H* = 7.87	*F* = 0.73	*F* = 1.38	*F* = 0.85	*F* = 0.91
*p*	0.232		0.698	0.581	0.447	0.605	0.207	0.560	0.513
*df*	8		8	8	8	5,137	8,197	8,197	8,197


*Summary of the nine groups’ mean age, mood prior to the experiment (MDBF short form A), art interest, trait emotional intelligence, empathy quotient and stress reactivity. *M*, mean; *SD*, standard deviation; *n*, number of participants; *p*, calculated probability; *df*, degrees of freedom; *H*, Kruskal–Wallis test statistic; *F*, *F*-test statistic; IAPS, International Affective Picture System; pos., positive; neg., negative; EI, emotional intelligence; EQ, empathy quotient; SRS, stress reactivity scale. Kruskal–Wallis tests conducted for the three conditions per picture set revealed no significant differences, except for a significant difference of trait EI between the three IAPS conditions, *H*(2) = 9.56, *p* = 0.008.*

#### Materials

**FIGURE 1:**
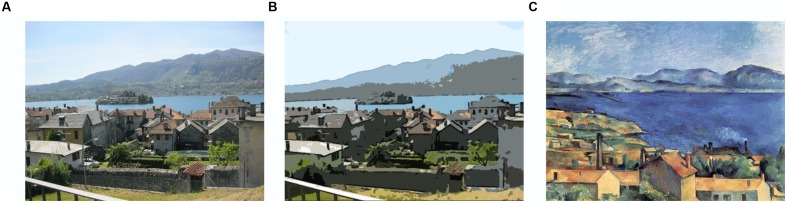
**Illustration of stimulus categories employed in Experiment 1.** **(A)** Photograph of a landscape (Manuela Marin), **(B)** photograph converted into a cartoon-like picture, and **(C)** representational painting by Paul Cézanne, *The bay of Marseille, seen from L’Estaque* (1885). Note that pictures **(A,B)** were not used in the actual experiment but are depicted for illustrative purposes only.

The 96 affective environmental scenes [taken from the International Affective Picture System (IAPS), Lang et al., 2005], and 96 representational paintings used in this experiment were identical to those used in Marin and Leder (2013, 2016). The pictures were in landscape format and varied in their semantic and emotional contents as well as in their complexity (figure-ground composition vs. complex scene). There were no pictures containing mutilation, erotic scenes or brand names. Marin and Leder (2016) showed that the subjective complexity ratings of these pictures are mostly determined by the number of objects, and to a lesser degree, by their disorganization. All scenes were converted into cartoon-like pictures using Adobe Photoshop CS5 and its filter functions (**Figure 1**). The first step involved the following processing stages: Filter-Artistic-Poster Edges (edge thickness = 0, edge intensity = 0, posterization = 1). Next, the Cutout filter was applied from the Photoshop filter gallery and the settings for number of levels, edge simplicity and edge fidelity were individually adjusted to obtain a picture whose semantic content could be easily deduced. In other words, the level of abstraction was not very high. Last, the brightness and contrast settings were adjusted (Image, Adjustment, brightness = 10, contrast = 30).

Standardized self-report measures included the two short forms A and B of the multidimensional mood questionnaire (MDBF, Steyer et al., 1997), the short trait emotional intelligence questionnaire (TEIQue-SF, Petrides and Furnham, 2006), the stress reactivity scale (SRS, Schulz et al., 2005) and a German version of the short Empathy Quotient (EQ-Short, Wakabayashi et al., 2006). For the groups rating representational paintings and cartoons, a short 15-item unpublished questionnaire on interest in visual arts was used. It comprised statements (developed in our research group), such as, “I enjoyed arts education at school,” “I am interested in art,” and “I don’t like ugly artworks,” and participants indicated their answers on 7-point scales ranging from “fully disagree” to “fully agree” (see Supplementary Table).

A self-developed questionnaire was administered after the performance of the experimental task. Participants were asked to report, on 7-point scales, how much they liked the pictures in general; how many they have seen before; whether they have experienced feelings while watching the pictures, and how strong these feelings were; whether they were emotionally engaged with the contents of the pictures; and how difficult it was to judge complexity. In the three conditions involving cartoons, participants also reported whether they would consider these pictures as art.

#### Procedure

We followed the current ethical guidelines at the University of Vienna and the version of the Ethical Principles of Psychologists and Code of Conduct of the American Psychological Association (with 2010 amendments). All participants read and signed an informed consent form prior to the experiment.

In general, the experimental procedure followed the one described in Experiments 1 and 2 in Marin and Leder (2013) and the one described in Experiment 1 in Marin and Leder (2016), but the order of the rating scales differed and standardized questionnaires were added. There were nine groups, which were defined by the type of picture set (environmental scenes, cartoons, and representational paintings) and the type of hedonic tone (beauty, pleasantness and liking). Participants filled the short form A of the multidimensional mood questionnaire before the actual experiment began. Then, sitting 60–70 cm away from the screen (19-inch Iiyama ProLite B1906S), participants were familiarized with the experimental task in two practice trials. Each trial was announced for 5 s and then a picture was shown on a black background for 5 s, after which the first (out of four) 7-point rating scale appeared on the screen, i.e., the one referring to hedonic tone. Ratings were given by mouse click and self-paced. Next, ratings of subjective arousal, complexity and familiarity were collected, after which the following trial was announced on the screen. Participants were asked to look at the picture for the entire presentation duration, and the picture was not shown when the ratings were collected. All pictures were blocked according to emotional contents (low-arousing pleasant, low-arousing unpleasant, high-arousing pleasant and high-arousing unpleasant) as pre-selected by Marin and Leder (2013), with an equal number of 23 trials in each block and a total number of 96 trials in each condition. The orders of the four blocks as well as the order of the pictures within each block were randomized. The participants were told that the blocks would vary in their emotional content, and they were allowed to take breaks between blocks.

The instructions for the measures of hedonic tone were as follows (note that only one of the scales was presented in each group of participants): Pleasantness: “Please rate the degree of pleasantness of your emotional experience,” with (1) “very unpleasant” and (7) “very pleasant” as anchors; Beauty: “Please report the degree of perceived beauty of the picture” with (1) “not at all beautiful” and (7) “very beautiful” as anchors; Liking: “Please report how much you liked the picture” with (1) “not at all” and (7) “very much” as anchors. Instructions for arousal ratings were “Please rate your felt arousal,” with (1) “very calm” and (7) “very excited” as anchors. Complexity was assessed by the following instruction: “Please rate your felt complexity of the picture,” with (1) “very simple” and (7) “very complex” as anchors. Finally, participants rated their familiarity with the picture content: “Please rate your familiarity with the contents of the picture,” with (1) “unfamiliar” and (7) “very familiar” as anchors.

After participants had finished the experiment, they filled the short form B of the multidimensional mood questionnaire, the self-developed questionnaire on the experiment, followed by the TeiQue-SF, SRS and EQ-short scales, and finally, the art interest scale (in the groups ratings cartoons and artworks). The experimental sessions lasted around 60 min. Participants were thanked, debriefed and dismissed.

#### Statistical Analysis

The analytical procedure regarding the analysis was identical to Marin and Leder (2013, 2016), thus enabling a meaningful comparison of the results across studies. The picture was chosen as the unit of analysis, which implies that the results need to be interpreted with regard to pictures and not participants. The main analysis was run using IBM SPSS Statistics 21. All statistical tests were computed at an alpha level of 0.05 and two-tailed. The Bonferroni–Holm procedure (Holm, 1979) was used to control the family wise error rate and computed in Matlab 2014b (The MathWorks Inc., Natick, MA, USA).

### Results

In a first step, the subjective ratings were averaged across participants for each picture in each condition. In a second step, the inter-rater reliability was assessed by calculating the average intra-class correlation coefficient (ICC) using a two-factor random effects model and type consistency (Shrout and Fleiss, 1979; McGraw and Wong, 1996). **Table 2** shows that the ICCs were generally high (all ICCs > 0.7), which justifies averaging across participants and considering pictures as unit of analysis. In a third step, outliers were determined by examining boxplots of each type of rating for each category of pictures. There were no outliers in the sets of IAPS pictures and cartoons. Familiarity and beauty ratings of paintings revealed two outliers, which were removed. Since most distributions were not normal, non-parametric analyses were employed to investigate the interrelationships between the four types of ratings in each condition.

**Table 2:** Intra-class correlation coefficients (ICCs) for subjective ratings of familiarity, complexity, arousal and hedonic tone (beauty, pleasantness and liking).

Group	ICC(2,k) Familiarity	ICC (2,k) Complexity	ICC (2,k) Arousal	ICC (2,k) Hedonic tone
IAPS Beauty	0.934	0.875	0.869	0.961
(*n* = 24)	CI [0.914,0.952]	[0.836,0.909]	[0.828,0.904]	[0.948,0.971]

IAPS Pleasantness	0.926	0.845	0.859	0.976
(*n* = 21)	CI [0.903,0.946]	[0.797,0.887]	[0.815,0.897]	[0.968,0.982]

IAPS Liking	0.937	0.884	0.900	0.963
(*n* = 23)	CI [0.917,0.954]	[0.848,0.915]	[0.868,0.927]	[0.952,0.973]

Cartoons Beauty	0.948	0.819	0.862	0.927
(*n* = 25)	CI [0.932,0.962]	[0.762,0.867]	[0.818,0.899]	[0.904,0.946]

Cartoons Pleasantness	0.927	0.836	0.852	0.969
(*n* = 22)	CI [0.904,0.947]	[0.785,0.880]	[0.805,0.891]	[0.960,0.978]

Cartoons Liking	0.941	0.818	0.887	0.957
(*n* = 24)	CI [0.922,0.956]	[0.761,0.867]	[0.851,0.917]	[0.943,0.968]

Paintings Beauty	0.764	0.836	0.824	0.891
(*n* = 22)	CI [0.690,0.828]	[0.785,0.880]	[0.768,0.871]	[0.856,0.920]

Paintings Pleasantness	0.765	0.867	0.814	0.964
(*n* = 20)	CI [0.691,0.828]	[0.826,0.903]	[0.756,0.864]	[0.952,0.973]

Paintings Liking	0.788	0.913	0.858	0.918
(*n* = 25)	CI [0.722,0.845]	[0.886,0.937]	[0.814,0.896]	[0.892,0.940]


*ICC(2,k), two-way random average measure, type consistency; CI, 95% confidence interval. ICCs were calculated for ratings of 96 pictures in each category (IAPS, cartoons and paintings).*

**Table 3** depicts the interrelationships between the four types of ratings, separately given for each of the nine conditions. Note that all relationships were visually inspected to ensure that they were monotonous. Familiarity with IAPS pictures and cartoons correlated negatively with complexity and arousal [ranging from small (*r*_s_ ∼0.2) to medium (*r*_s_ ∼0.3) effect sizes], with stronger relationships for cartoons than IAPS pictures. The relationships between familiarity and measures of hedonic tone were positive, strong, and constant across the three measures of hedonic tone (*r*_s_ > 0.6), and comparable in IAPS pictures and cartoons. A similar pattern of results was observed for paintings; however, there was more variation across the three measures of hedonic tone in terms of effect size.

**Table 3:** Spearman’s rank order correlations between measures of familiarity, complexity, arousal and measures of hedonic tone in response to IAPS pictures, cartoons and paintings.

Stimulus type	Group	Measure	Familiarity	Complexity
IAPS	Beauty	Complexity	-0.19	
	Pleasantness		-0.19	
	Liking		-0.29*	
	Beauty	Hedonic tone	0.66*	-0.05
	Pleasantness		0.62*	-0.15
	Liking		0.62*	-0.24*
*N* = 96	Beauty	Arousal	-0.30*	0.34*
*df* = 94	Pleasantness		-0.24	0.29*
	Liking		-0.19	0.40*

Cartoons	Beauty	Complexity	-0.33*	
	Pleasantness		-0.31*	
	Liking		-0.36*	
	Beauty	Hedonic tone	0.68*	-0.13
	Pleasantness		0.61*	-0.29*
	Liking		0.62*	-0.10
*N* = 96	Beauty	Arousal	-0.44*	0.34*
*df* = 94	Pleasantness		-0.27*	0.30*
	Liking		-0.34*	0.45*

Paintings	Beauty	Complexity	0.09	
	Pleasantness		-0.16	
	Liking		0.11	
	Beauty	Hedonic tone	0.64*	0.25*
	Pleasantness		0.71*	-0.22
	Liking		0.59*	0.08
*N* = 94	Beauty	Arousal	-0.28*	0.42*
*df* = 92	Pleasantness		-0.52*	0.47*
	Liking		-0.29*	0.43*


*^∗^*p* < 0.05 after Bonferroni–Holm correction; *df*, degrees of freedom.*

Controlling for familiarity effects, **Table 4** shows how complexity, arousal and the three measures of hedonic tone were related. In line with Berlyne’s prediction, we generally observed small to moderate associations between complexity and arousal for both IAPS pictures and cartoons. In paintings, this association was somewhat stronger (*r*_s_ ∼0.5), nearly reaching the benchmark of a large effect (Cohen, 1988), supporting our prediction. Furthermore, the data suggested that the relationship between complexity and arousal was the strongest in the liking condition, regardless of the picture type.

**Table 4:** Partial Spearman’s rank order correlations controlling for familiarity between measures of complexity, arousal and hedonic tone in response to IAPS pictures, cartoons and paintings.

Stimulus type	Group	Measure	Complexity	Hedonic tone
IAPS	Beauty	Hedonic tone	0.10	
	Pleasantness		-0.04	
	Liking		-0.08	
*N* = 96	Beauty	Arousal	0.30**	N/A
*df* = 93	Pleasantness		0.26*	N/A
	Liking		0.37***	N/A

Cartoons	Beauty	Hedonic tone	0.13	
	Pleasantness		-0.14	
	Liking		0.16	
*N* = 96	Beauty	Arousal	0.23*	N/A
*df* = 93	Pleasantness		0.24*	N/A
	Liking		0.37***	N/A

Paintings	Beauty	Hedonic tone	0.26*	
	Pleasantness		-0.16	
	Liking		0.02	
*N* = 94	Beauty	Arousal	0.46***	-0.07
*df* = 91	Pleasantness		0.46***	-0.56***
	Liking		0.49***	-0.23*


*p < 0.05, ^∗∗^*p* < 0.01, ^∗∗∗^*p* < 0.001; *df*, degrees of freedom. The relationships between the respective measures of hedonic tone and arousal were quadratic in IAPS pictures and cartoons and are thus not reported.

**FIGURE 2:**
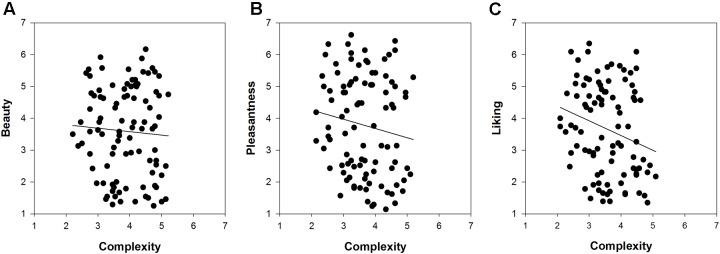
**The relationships between complexity and the three measures of hedonic tone for 96 IAPS pictures: **(A)** beauty, **(B)** pleasantness, and **(C)** liking**.

**FIGURE 3:**
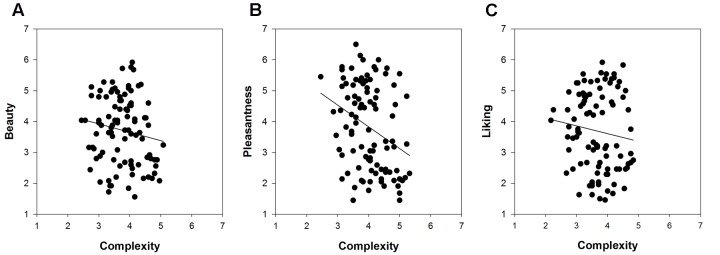
**The relationships between complexity and the three measures of hedonic tone for 96 cartoons based on IAPS pictures: **(A)** beauty, **(B)** pleasantness, and **(C)** liking**.

**FIGURE 4:**
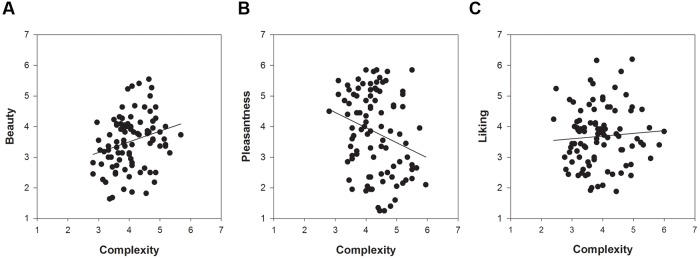
**The relationships between complexity and the three measures of hedonic tone for 94 representational paintings: **(A)** beauty, **(B)** pleasantness, and **(C)** liking**.

Of particular interest was the comparison of the relationships between complexity and the respective measures of hedonic tone across the three picture sets. **Figures 2**–**4 (A–C)** show these relationships including a regression line based on results of curve-fitting analyses conducted in SigmaPlot 13.0. By not controlling for familiarity effects, the multifaceted nature of hedonic tone became visually apparent in cartoons and paintings but not in IAPS pictures. The relationship between complexity and pleasantness was negative in cartoons, whereas there were no relationships for the beauty and liking conditions. In paintings, the relationships differed to a much larger extent and the graphs seemed to indicate a positive association between complexity and beauty, a negative association between complexity and pleasantness, and no relationship in the liking condition.

Turning to the results of partial correlations controlling for familiarity effects, we predicted a positive relationship for beauty based on results by Nadal et al. (2010). In IAPS pictures and cartoons we found weak indications for a positive association, but these relationships were not significant (**Table 4**). The relationship between complexity and beauty was positive and significant only for paintings (*r*_s_ = 0.26). Next, we predicted no relationship between complexity and pleasantness, which held true for IAPS pictures. For cartoons and paintings we observed indications of a weak, non-significant negative relationship. The relationships between complexity and liking ratings were absent in IAPS pictures and paintings, but weakly non-significantly positive in cartoons. We also compared the correlation coefficients expressing the relationship between complexity and hedonic tone by means of a Fisher *r*-to-*z* transformation in paintings. The coefficients of the beauty-complexity and pleasantness-complexity relationships significantly differed from each other (*p* = 0.004), whereas the beauty-complexity and liking-complexity relationships (*p* = 0.097) as well as the pleasantness-complexity and liking-complexity relationships (*p* = 0.223) were not significantly different form each other. Taken together, these results show that the relationship between complexity and hedonic tone depends on the specification of the latter concept (i.e., beauty, pleasantness and liking), which became mostly apparent in the study of paintings.

Due to the pre-selection of the stimuli, the relationships between the respective measures of hedonic tone and arousal were quadratic in IAPS pictures (see Marin and Leder, 2013, 2016) and cartoons and did not change much across the three conditions of hedonic tone. However, two of these relationships were linear in paintings (**Table 4**): we observed negative relationships for pleasantness (*r*_s_ = -0.56) and liking (*r*_s_ = -0.23) but not for beauty, providing more evidence for the multifaceted nature of hedonic tone in the perception of paintings.

If hedonic tone is not a uniform concept when considering the perception of paintings, intercorrelations between the different measures of hedonic tone should be lower for ratings of paintings than for IAPS pictures and cartoons. In IAPS pictures (*N* = 96), the correlation strengths between measures of hedonic tone were very high: *r*_beauty-pleasantness_ = 0.91, *r*_beauty-liking_ = 0.95, and *r*_liking-pleasantness_ = 0.92. In cartoons (*N* = 96), these relationships were similar, but the beauty-pleasantness relationship was slightly lower: *r*_beauty-pleasantness_ = 0.85, *r*_beauty-liking_ = 0.90, and *r*_liking-pleasantness_ = 0.92. However, in paintings (*N* = 94), the three measures correlated to a lesser extent with each other: *r*_beauty-pleasantness_ = 0.73, *r*_beauty-liking_ = 0.91, and *r*_liking-pleasantness_ = 0.77. Overall the pattern of results suggests that the relationship between beauty and pleasantness as well as the one between liking and pleasantness were weaker for paintings than for the other two stimulus sets.

Intercorrelations between the three complexity ratings of each picture set revealed that they were generally highly correlated (subscripts refer to the three conditions per picture set): IAPS pictures: *r*_beauty-pleasantness_ = 0.90, *r*_beauty-liking_ = 0.85, and *r*_liking-pleasantness_ = 0.87; cartoons: *r*_beauty-pleasantness_ = 0.84, *r*_beauty-liking_ = 0.87, and *r*_liking-pleasantness_ = 0.80; paintings: *r*_beauty-pleasantness_ = 0.85, *r*_beauty-liking_ = 0.85, and *r*_liking-pleasantness_ = 0.88. For arousal ratings, these intercorrelations across the three conditions per picture set were of similar strength: IAPS pictures: *r*_beauty-pleasantness_ = 0.85, *r*_beauty-liking_ = 0.90, and *r*_liking-pleasantness_ = 0.87; cartoons: *r*_beauty-pleasantness_ = 0.81, *r*_beauty-liking_ = 0.87, and *r*_liking-pleasantness_ = 0.82; paintings: *r*_beauty-pleasantness_ = 0.81, *r*_beauty-liking_ = 0.83, and *r*_liking-pleasantness_ = 0.85.

The mean complexity ratings of all pictures per type of category and condition were as follows: IAPS pictures: *M*_beauty_ = 3.87, *SD*_beauty_ = 0.77, *M*_pleasantness_ = 3.65, *SD*_pleasantness_ = 0.77, *M*_liking_ = 3.55, *SD*_liking_ = 0.74; cartoons: *M*_beauty_ = 3.76, *SD*_beauty_ = 0.61, *M*_pleasantness_ = 4.02, *SD*_pleasantness_ = 0.63, *M*_liking_ = 3.66, *SD*_liking_ = 0.61; paintings: *M*_beauty_ = 4.05, *SD*_beauty_ = 0.63, *M*_pleasantness_ = 4.31, *SD*_pleasantness_ = 0.68, *M*_liking_ = 3.90, *SD*_liking_ = 0.78.

The analysis of the post-experimental questionnaire (**Table 5**) showed that participants’ mean familiarity ratings were generally low (mean ratings around 2 across conditions on the 7-point rating scale). Furthermore, participants’ liking ratings were neutral on average, which was to be expected given that pictures of different emotional contents were shown. In all conditions, participants reported to have felt feelings (mean ratings around 5 across conditions on the 7-point rating scale), which were of an average intensity. Moreover, participants also reported moderate emotional involvement with the picture content in all conditions. The difficulty to judge complexity was neutral across conditions. Ratings of the artistic status of cartoons showed that participants rated them above average (around 4.2 on a 7-point rating scale). A comparison across the three picture types by means of a series of Kruskal–Wallis tests revealed that participants neither preferred one picture type to the other nor did they report different levels of emotional involvement. All picture types induced emotions of similar intensity. However, the data suggest that reporting complexity judgments was somewhat easier for paintings than for the other two picture types, *H*(2) = 5.70, *p* = 0.058. General familiarity ratings were higher for cartoons than for the other two picture sets, *H*(2) = 6.35, *p* = 0.042, which was due to higher familiarity ratings in the condition in which participants reported liking of cartoons.

**Table 5:** Post-experiment questionnaire.

Group	General Liking		General Familiarity	Frequency of feelings	Intensity of feelings	Empathy	Difficulty to judge complexity	Artistic quality of cartoons
IAPS Beauty	*M*	4.17	1.83	5.33	4.50	5.50	4.63	–
(*n* = 24)	*SD*	1.37	1.17	1.24	1.14	1.14	1.38

IAPS Pleasantness	*M*	4.71	2.67	5.52	5.05	5.86	4.95	–
(*n* = 21)	*SD*	1.31	1.39	0.75	0.81	0.73	1.60

IAPS Liking	*M*	4.35	2.70	4.74	4.22	5.39	4.91	–
(*n* = 23)	*SD*	1.53	1.33	0.96	1.41	1.27	1.44

Cartoons Beauty	*M*	3.88	2.48	4.76	3.88	5.28	4.44	4.20
(*n* = 25)	*SD*	1.59	1.39	1.42	1.24	1.37	1.71	1.66

Cartoons Pleasantness	*M*	4.18	2.73	5.00	4.41	5.41	4.55	4.27
(*n* = 22)	*SD*	1.56	1.98	1.02	1.37	0.80	1.47	1.80

Cartoons Liking	*M*	4.04	3.83	4.71	4.13	5.17	4.92	4.21
(*n* = 24)	*SD*	1.43	1.86	1.23	1.57	1.20	1.59	1.64

Paintings Beauty	*M*	3.95	2.05	4.5	3.91	5.14	3.73	–
(*n* = 22)	*SD*	1.36	1.00	1.26	1.80	1.49	1.39

Paintings Pleasantness	*M*	4.75	2.35	5.25	4.60	5.80	4.00	–
(*n* = 20)	*SD*	1.56	1.31	1.07	1.05	0.77	1.32

Paintings Liking	*M*	4.32	2.08	4.92	4.00	5.40	4.84	–
(*n* = 25)	*SD*	1.31	0.95	1.38	1.56	1.23	1.72


*Summary of the nine groups’ mean answers to questions referring to general aspects of the experiment. *M*, mean; *SD*, standard deviation; *n*, number of participants; General liking: “How much did you like the pictures in general?”; General familiarity: “Please estimate how many pictures you have seen prior to the experiment.”; Frequency of feelings: “Did you experience any feelings while looking at the pictures?”; Intensity of feelings: “How strong were the feelings evoked by the pictures?”; Empathy: “Did you feel emotionally involved with the visual contents of the pictures?”; Difficulty to judge complexity: “How difficult was it to rate the complexity of the pictures?”; Artistic quality of cartoons: “Would you consider these pictures as art?”*

### Discussion

We studied the relationship between complexity and three measures of hedonic tone in three sets of visual stimuli, namely environmental scenes, environmental scenes converted into cartoons, as well as representational paintings. In general, the current findings corroborated our hypotheses: After partialling out familiarity effects, we showed that visual complexity related to the number and diversity of elements and arousal were related in all three picture sets, and further, that this relationship was strongest for representational paintings, reaching a moderate effect size. The data also revealed that the multifaceted nature of hedonic tone was only clearly present during the perception of paintings: although the relationship between complexity and arousal was the same in all three conditions of hedonic tone, the relationship between complexity and hedonic tone differed across conditions. In line with Nadal et al. (2010), we observed a positive relationship between complexity and beauty. As predicted based on results by Marin and Leder (2016), we did not detect a significant relationship between complexity and pleasantness, although there were indications of a negative association between these measures if familiarity effects were ignored. Complexity and liking did not correlate with each other.

Further support for the multifaceted nature of hedonic tone in relation to paintings stems from the fact that the strength of the relationship between arousal and the three measures of hedonic tone differed considerably. The negative correlation was strongest between pleasantness and arousal, followed by the one between liking and arousal, and interestingly, there was no relationship between beauty and arousal. The relationship between the specific measure of hedonic tone and complexity during the experience of affective, representational human artworks may thus relate to hedonic measures’ differential effects on arousal, a core component of Berlyne’s theory. Moreover, the strength of the positive intercorrelations between the three measures of hedonic tone was lowest in paintings, although it was generally still high.

Marin and Leder (2016) investigated subjective responses to the same set of affective environmental scenes and representational paintings as used in the current study. Since the presentation duration of the pictures (5 s), the participant sample, as well as one measure of hedonic tone, namely pleasantness, were similar in the two studies, a detailed comparison of results is meaningful. The current results closely replicate findings by Marin and Leder (2016), who reported a correlation of *r*_s_ = 0.27 for the relationship between complexity and arousal in IAPS pictures and a correlation of *r*_s_ = 0.52 for the same relationship in paintings. Moreover, the current findings are in line with the weakly negative relationship (*r*_s_ = -0.15) between complexity and pleasantness observed for paintings. However, Marin and Leder (2016) reported a weak positive relationship between complexity and pleasantness for IAPS pictures (*r*_s_ = 0.22), which was not supported by the current data, and also not by Marin and Leder (2013). It should be noted that Marin and Leder (2016) did not find a relationship between complexity and pleasantness for the presentation durations of 1 and 25 s, so we regard this finding as a possible random outcome. Altogether, the comparison of results across the two studies indicates that the nature of the relationship between complexity and arousal seems to be robust for these affective stimuli sets in groups of female participants.

Our findings are in line with previous studies employing more than one measure of hedonic tone, suggesting that people differentiate more between concepts such as beauty, pleasantness and liking during the perception of paintings (Berlyne and Ogilvie, 1974; Russell and George, 1990) than during the perception of real-life scenes or abstract visual patterns (Normore, 1974; Imamoglu, 2000). Here, we extend these findings by employing a strictly controlled research design that directly compares subjective responses across different stimulus sets within the same experimental framework. However, it still remains to be studied whether the multifaceted nature of hedonic tone and its relation to complexity is specific to esthetic experiences of human made artworks. The current data suggest that ratings of beauty, pleasantness and liking are nearly identical in the perception of affective and motivationally relevant environmental scenes, which probably have not evoked esthetic experiences. Therefore, our results motivate the systematic, comparative study of other stimulus categories in the visual domain.

The current results regarding the comparison across different picture sets are relevant to neuroimaging studies reporting brain areas being specifically active during the perception of art. Several neuroimaging studies have recently followed a comparative approach and investigated brain regions active during the perception of art and non-art images matched for semantic contents (Di Dio et al., 2011; Lacey et al., 2011; Lutz et al., 2013; Mizokami et al., 2014). Di Dio et al. (2011) investigated neural responses to masterpieces of classical masters and matched photographs of young athletes. In general, the activation patterns for the two types of stimuli were very similar; however, only artistic stimuli activated the right dorsal anterior insula, a brain area that has been found to link emotion and cognition. In a similar vein, Lutz et al. (2013) examined body representations in paintings and matched non-artistic photographs of body parts. The results indicated that processing of paintings was accompanied by the activation of the right parietal cortex and the extrastriate cortex bilaterally, which led the authors to conclude that the experience of visual art is a distinct perceptual process. Lacey et al. (2011) carefully matched photographs of everyday life with paintings and asked for several types of ratings (beauty, liking and pleasantness) after the fMRI scanning. The authors found activation of the ventral striatum for paintings but not for photographs. Interestingly, the authors did not report activation of the amygdala, and further, did not report correlations between the brain activity patterns and subjective ratings of beauty, pleasantness and liking. This suggests that activation of the ventral striatum may be related to the status of images as art and not to the esthetic experience itself as measured by subjective ratings. Mizokami et al. (2014) created visual scenes that very closely representing the semantic content of the landscapes and still life’s and reported no correlation between beauty ratings and brain activations. Contrasting activations for paintings and photographs showed activations of the bilateral cuneus and the left lingual gyrus.

These previous studies comparing the neural correlates of different visual stimulus categories have largely ignored the affective contents of the stimuli and mostly focused on the matching of the semantic contents between categories. If one decided to follow an affective approach to the study of visual art, it would be crucial to incorporate pleasantness and arousal in the research design and stimulus selection, given that distinct neural networks related to the processing of pleasant and unpleasant environmental scenes (IAPS pictures) have been described (Aldhafeeri et al., 2012). The processing of pleasant pictures yielded significant activation in the bilateral prefrontal cortex, including the superior, medial and middle frontal gyri. Other active brain regions comprised the right anterior and posterior cingulate gyri and both temporal lobes. Unpleasant pictures elicited bilateral activation in the amygdala, hippocampus, parahippocampal gyri as well as secondary and primary visual cortex. These neural networks could be taken as a starting point for a neuroimaging study employing representational paintings. We further propose that future neuroimaging studies comparing different stimulus categories may also consider structural features of visual stimuli, such as aspects of complexity, and their relation to measures of hedonic tone. We would hypothesize that distinct activation patterns may emerge in the perception of paintings in comparison to the perception of matched non-art images. Such an approach would also contribute to a better understanding of the complex interplay between cognition and emotion during esthetic experiences.

## Experiment 2

### Materials and Methods

#### Participants

Participants were 92 female psychology students of the University of Vienna, all non-musicians (less than 3 years of musical training in the past and no musical activity at the time of the experiment). Participants were randomly assigned to one of three groups differing in the type of hedonic tone to be rated (beauty, pleasantness and liking). Participants were screened for outliers with respect to their mood prior to the experiment (participants with very low mood scores were removed), their musical sophistication as well as the trait emotional intelligence, emotional self-efficacy and SRSs. Fifteen participants were removed for these reasons by investigating the respective boxplots. The remaining 77 participants in the three groups did not significantly differ regarding their age, mood prior to the experiment and their scores on the trait EI, emotional self-efficacy and SRS. We also ensured that musical sophistication was similar and generally low (see **Table 6**).

**Table 6:** Participant characteristics.

Group	Age [years]		Pos./neg. mood A	Alertness/fatigue A	Quietude/disquietude A	Gold-MSI	Trait EI	ES	SRS
Beauty	*M*	21.0	16.31	12.54	15.73	56.00	144.27	86.00	62.38
(*n* = 26)	*SD*	1.8	2.24	2.61	2.82	10.80	19.18	10.11	9.83

Pleasantness	*M*	20.5	16.72	13.64	15.20	53.24	146.60	86.04	60.92
(*n* = 25)	*SD*	1.4	2.28	2.87	2.75	14.58	15.14	8.52	7.76

Liking	*M*	21.8	17.54	12.62	15.00	53.88	146.96	84.23	61.27
(*n* = 26)	*SD*	2.8	1.68	3.29	3.03	15.25	17.51	10.33	6.26

Test statistic	*H* = 1.85		*H* = 4.30 2	*H* = 1.89	*H* = 1.02	*F* = 2.89	*F* = 0.18	*F* = 0.29	*F* = 0.23
*p*	0.396		0.116	0.389	0.601	0.752	0.833	0.747	2,74
*df*	2		2	2	2	2,74	2,74	2,74	2,74


*Summary of the three groups’ mean age, mood prior to the experiment (MDBF short form A), musical sophistication, trait emotional intelligence, emotional self-efficacy and stress reactivity. *M*, mean; *SD*, standard deviation; *n*, number of participants; *p*, calculated probability; *df*, degrees of freedom; *H*, Kruskal–Wallis test statistic;*F*, *F*-test statistic; Gold-MSI, Goldsmiths Musical Sophistication Index; pos., positive; neg., negative; EI, emotional intelligence; ES, emotional self-efficacy; SRS, stress reactivity scale.*

#### Materials

Ninety-two musical excerpts, taken from Experiment 3 of Marin and Leder (2013), were shortened to a duration of 15 s by removing the endings of the original excerpts. A fade-out (500 ms) was added to the excerpts using Audacity 2.0.6 software. These musical stimuli varied naturally in complexity and were pre-selected to cover the two-dimensional emotion space spanned by pleasantness and arousal (Russell, 1980).

As in Experiment 1, several standardized self-report measures were administered, including the two short forms A and B of the multidimensional mood questionnaire (MDBF, Steyer et al., 1997), the short trait emotional intelligence questionnaire (TEIQue-SF, Petrides and Furnham, 2006), the stress reactivity scale (SRS, Schulz et al., 2005), and the emotional self-efficacy scale by Schmitz and von Salisch (2002). Musical sophistication was assessed with the German version of the Goldsmiths Musical Sophistication Index (Gold-MSI) developed by Schaal et al. (2014).

A self-developed questionnaire comprised a set of questions to be answered on 7-point scales referring to the general liking of the musical excerpts, the difficult of judging musical complexity and arousal, and the strengths of the feelings induced by the music. Participants were also asked to report on the role of music in their lives and preferences for different musical styles.

#### Procedure

As in Experiment 1, we followed the current ethical guidelines at the University of Vienna and the version of the Ethical Principles of Psychologists and Code of Conduct of the American Psychological Association (with 2010 amendments). All participants read and signed an informed consent form.

Participants filled in the short form A of the multidimensional mood questionnaire prior to the experiment. The experimental setting was identical to the one described in Experiment 1 except that musical stimuli were played through an external soundcard (E-MU audio interface, 0204/USB) and participants were wearing Sennheißer HD 380 pro headphones. The volume was fixed to approximately 72 dB SPL (A-weighted) as measured during the presentation of the second practice trial (see Marin and Leder, 2013). Sitting 60–70 cm away from the screen (19-inch Iiyama ProLite B1906S), participants were familiarized with the experimental task in two practice trials. Each trial was announced for 5 s and then the excerpt was played for 15 s, after which the first (out of four) 7-point rating scale appeared on the screen (familiarity, complexity, arousal and hedonic tone). The instructions were identical as those used in Experiment 1. Familiarity ratings referred to the familiarity with the musical excerpt. All ratings were given by mouse click and self-paced.

The musical excerpts were blocked according to emotional contents (low-arousing pleasant, low-arousing unpleasant, high-arousing pleasant and high-arousing unpleasant). The order of the four blocks as well as the order of the 23 excerpts within each block were randomized. The participants were told that the blocks would vary in their emotional content, and they were allowed to take breaks between blocks. After the experiment, participants filled in the short form B of the mood questionnaire, and then the other questionnaires in one of two different orders (TEIQue-SF, SRS, emotional self-efficacy, and Gold-MSI or emotional self-efficacy, SRS, TEIQue-SF, and Gold-MSI). The experimental session lasted around 90 min, after which participants were debriefed, thanked and dismissed.

### Results

The analysis of the data follows the one presented in Experiment 1. The subjective ratings were averaged across participants for each of the musical excerpt in each condition. Then we assessed the inter-rater reliability by calculating the average intra-class correlation coefficient (ICC) using a two-factor random effects model and type consistency (Shrout and Fleiss, 1979; McGraw and Wong, 1996). **Table 7** shows that the ICCs were generally high (all ICCs > 0.7), which justifies averaging across participants and considering excerpts as unit of analysis. Next, outliers were determined by examining boxplots for each type of rating for each condition. There were no outliers regarding familiarity, complexity and arousal ratings. One excerpt was removed due to very low pleasantness ratings, four excerpts due to very low beauty ratings, and one due to very low liking ratings. Since most distributions were not normal, non-parametric analyses were employed to investigate the interrelationships between the four types of ratings in each condition.

**Table 7:** Intra-class correlation coefficients (ICCs) for subjective ratings of familiarity, complexity, arousal and hedonic tone (beauty, pleasantness and liking).

Group	ICC(2,k) Familiarity	ICC(2,k) Complexity	ICC(2,k) Arousal	ICC(2,k) Hedonic tone
Beauty	0.717	0.897	0.923	0.810
(*n* = 26)	CI [0.626,0.794]	[0.864,0.925]	[0.898,0.944]	[0.749,0.862]

Pleasantness	0.742	0.934	0.926	0.851
(*n* = 25)	CI [0.659,0.812]	[0.913,0.952]	[0.903,0.946]	[0.804,0.892]

Liking	0.776	0.952	0.935	0.820
(*n* = 26)	CI [0.705,0.837]	[0.936,0.965]	[0.915,0.953]	[0.763,0.869]


*ICC(2,k), two-way random average measure, type consistency; CI, 95% confidence interval. ICCs were calculated for ratings of 92 musical excerpts.*

**FIGURE 5:**
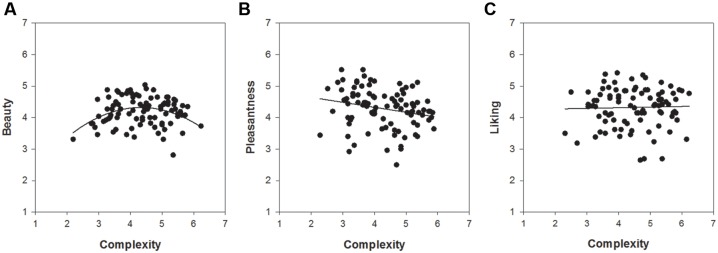
**The relationships between complexity and the three measures of hedonic tone for 19th-century piano solo music: **(A)** beauty, **(B)** pleasantness, and **(C)** liking**.

**Table 8** depicts the interrelationships between the four types of ratings, separately given for each of the three conditions. All relationships were visually inspected to ensure that they were monotonous before Spearman’s rank correlations were computed. Familiarity was only mildly negatively associated with complexity and arousal, whereas the relationships between familiarity and measures of hedonic tone were positive, ranging from medium (*r*_s_ ∼0.53) to strong effect sizes (*r*_s_ ∼0.75). The relationship between complexity and arousal was strong and of equal strength in all three conditions of hedonic tone (*r*_s_ ∼0.85). The relationship between complexity and beauty was not linear (**Figure 5**). The relationship followed an inverted-U curve and curve-fitting analysis confirmed its quadratic nature, *F*(2,85) = 5.24, *p* = 0.007, *R^2^* = 0.11, y = 0.9 + 1.58x–0.18x^2^. Complexity and pleasantness were significantly negatively associated (*r*_s_ ∼-0.25), and the data revealed no relationship between complexity and liking.

**Table 8:** Spearman’s rank order correlations between measures of familiarity, complexity, arousal and measures of hedonic tone in response to 19th-century piano solo music.

Group	*df*	Measure	Familiarity	Complexity
Beauty	86	Complexity	0.10	
Pleasantness	89		-0.08	
Liking	89		-0.21	

Beauty	86	Hedonic tone	0.53^∗^	N/A
Pleasantness	89		0.64^∗^	-0.25^∗^
Liking	89		0.75^∗^	0.04

Beauty	86	Arousal	0.003	0.86^∗^
Pleasantness	89		-0.30^∗^	0.85^∗^
Liking	89		-0.35^∗^	0.85^∗^


*p < 0.05 after Bonferroni–Holm correction; *df*, degrees of freedom.

Controlling for familiarity effects, **Table 9** shows how complexity, arousal and the three measures of hedonic tone were associated. The strong and positive association between complexity and arousal is in line with Berlyne’s theory. Three different relationships between complexity and the respective measures of hedonic tone emerged: complexity and pleasantness were negatively associated (*r*_s_ ∼-0.26), complexity and liking positively (*r*_s_ ∼0.29), and finally, complexity and beauty ratings followed an inverted-U curve as predicted by Berlyne. The two linear relationships significantly differed from each other, *z* = -3.72, *p* = 0.0002. Next, a hierarchical linear regression analysis was performed to control for effects of familiarity on beauty ratings and to demonstrate the quadratic nature of the relationship. Familiarity was entered in the first block, followed by adding the linear term for complexity ratings, and finally, the quadratic term for complexity was entered in the third step. The results of the final model were significant, *F*(3,87) = 17.31, *p* < 0.001, adjusted *R^2^* = 0.36, and revealed the following significant predictors: familiarity (β = 0.54, *p* < 0.001), complexity (β = 1.72, *p* = 0.041) and complexity squared (β = -2.12, *p* = 0.037).

**Table 9:** Partial Spearman’s rank order correlations controlling for familiarity between measures of complexity, arousal and hedonic tone in response to 19th-century piano solo music.

Group	*df*	Measure	Complexity	Hedonic tone
Beauty	85	Hedonic tone	N/A	
Pleasantness	88		-0.26^∗^	
Liking	88		0.29^∗∗^	

Beauty	85	Arousal	0.87^∗∗∗^	N/A
Pleasantness	88		0.87^∗∗∗^	-0.44^∗∗∗^
Liking	88		0.85^∗∗∗^	0.24^∗^


*p < 0.05, ^∗∗^*p* < 0.01, ^∗∗∗^*p* < 0.001; *df*, degrees of freedom.

**Table 9** also shows that the relationships between arousal and measures of hedonic tone differed from each other. The relationship between arousal and beauty was of a non-monotonic quadratic nature, though curve-fitting analysis did not reveal a significant trend. The relationship between pleasantness and arousal was negative (*r*_s_ ∼-0.44), and the one between arousal and liking positive (*r*_s_ ∼0.24). In general, the correlations between arousal and measures of hedonic tone reflect the nature of the complexity-hedonic tone relationship.

Next, intercorrelations between measures of hedonic tone were computed (*N* = 88), with the lowest correlation between liking and pleasantness ratings: *r*_beauty-pleasantness_ = 0.70, *r*_beauty-liking_ = 0.76, and *r*_liking-pleasantness_ = 0.59. Intercorrelations between the three complexity ratings of the three conditions were generally very high: *r*_beauty-pleasantness_ = 0.96, *r*_beauty-liking_ = 0.96, and *r*_liking-pleasantness_ = 0.96. For arousal ratings, these intercorrelations across the three conditions were of similar strength: *r*_beauty-pleasantness_ = 0.94, *r*_beauty-liking_ = 0.95, and *r*_liking-pleasantness_ = 0.95. The mean complexity ratings of the three conditions were as follows: *M*_beauty_ = 4.35, *SD*_beauty_ = 0.88, *M*_pleasantness_ = 4.27, *SD*_pleasantness_ = 0.91, *M*_liking_ = 4.49, *SD*_liking_ = 0.94.

Participants also answered several questions referring to the experiment (**Table 10**). The three groups did not differ regarding their general liking of the musical excerpts, their familiarity with them, the frequency and strength of reported feelings as well as the difficulty of judging musical complexity. Significant group differences were observed for the role of music in participants’ life, *H*(2) = 8.64, *p* = 0.013, as well as for the frequency of listening to classical music, *H*(2) = 7.73, *p* = 0.021. Participants in the beauty group reported a higher role of music in their lives and a lower frequency of listening to music in comparison to the other two groups.

**Table 10:** Post-experiment questionnaire.

Group	General Liking		General Familiarity	Frequency of feelings	Intensity of feelings	Difficulty to judge complexity	Role of music in life	Listening to classical music
Beauty	*M*	4.46	2.34	4.77	3.88	4.65	5.88	2.15
(*n* = 26)	*SD*	1.45	0.80	1.39	1.48	1.62	0.86	1.08

Pleasantness	*M*	4.88	3.08	4.72	3.68	5.04	5.12	2.76
(*n* = 25)	*SD*	1.09	1.08	1.14	0.95	1.37	1.17	1.30

Liking	*M*	5.08	2.77	4.50	3.69	4.23	5.00	3.23
(*n* = 26)	*SD*	1.55	1.50	1.50	1.59	1.86	1.26	1.48


*Summary of the three groups’ mean answers to questions referring to general aspects of the experiment. *M*, mean; *SD*, standard deviation; *n*, number of participants; General liking: “How much did you like the music in general?”; General familiarity: “Please estimate how many musical excerpts you have heard prior to the experiment.”; Frequency of feelings: “Did you experience any feelings while listening to the music?”; Intensity of feelings: “How strong were the feelings evoked by the music?”; Difficulty to judge complexity: “How difficult was it to rate the complexity of the musical excerpts?”, Role of music in life: “What role does music play in your life?”*

### Discussion

We explored the relationship between musical complexity and three measures of hedonic tone (beauty, pleasantness and liking) in a highly controlled participant sample and between-subject design. The same stimulus set was rated for familiarity, complexity, arousal and one of the three measures of hedonic tone. We observed that the nature of the relationship differed across groups when controlling for familiarity effects. Not supporting our hypotheses, but in line with Berlyne’s theory (Berlyne, 1971), our data indicated an inverted-U relationship between complexity and beauty ratings. Moreover, we observed a weak negative relationship between complexity and pleasantness ratings and a weak positive relationship between complexity and liking. In other words, all three measures of hedonic tone showed different relationships with complexity. Arousal and complexity were highly correlated, as predicted by Berlyne, and the effect size was not only nearly identical across the three groups, but also very similar to results reported in Experiment 3 in Marin and Leder (2013).

Marin and Leder (2013) studied the relationship between complexity and pleasantness using longer versions of the musical excerpts employed in Experiment 2. After controlling for familiarity effects, their results indicated no relationship between complexity and pleasantness in females, but a positive relationship between these measures in males. Here, we found a negative relationship between complexity and pleasantness. Because we employed a more rigorous approach during participant sampling than in Marin and Leder (2013), it may be that other factors such as mood or musical sophistication influenced previously reported results.

The present results further show that the relationship between arousal and measures of hedonic tone is comparable to the respective relationships between complexity and hedonic tone (see **Table 9**), lending more support to Berlyne’s theory regarding the crucial role of arousal in esthetic experiences. This finding was also reported in Marin and Leder (2013) and thus clearly suggests that arousal plays a key role in the complexity-hedonic tone relationship. Future research may investigate larger samples of participants in order to test whether arousal acts as a mediator in the complexity-hedonic tone relationships. Using stimuli as the unit of analysis, Marin and Leder (2013) already provided some support for Berlyne’s theory by testing mediation with the help of regression analysis. However, it may be more appropriate to test mediation using individual participant data, which requires a much larger sample than the one tested in this study.

The stimulus selection in Marin and Leder (2013) primarily focused on the emotional content of the stimuli, following Russell’s (1980) circumplex model of affect, and no particular attention was paid to the complexity of the musical excerpts. This stands in contrast to the approach presented in Experiment 1, where complexity was considered during the stimulus selection and differed mostly along the dimension of elements (Marin and Leder, 2016). Since it is not known yet whether the perception of musical complexity can be understood using dimensions comparable to those in the visual domain, such as number and diversity of elements or disorganization, a direct comparison between the specific outcomes for each of the hedonic measures across the visual and musical domains must be made with caution. However, our findings strongly suggest that in the appreciation of paintings and music, the nature of the complexity-hedonic tone relationship depends on the specific measure of hedonic tone if complexity is held constant.

## General Discussion

We sought to contribute to a better understanding of the discrepant research results regarding Berlyne’s (1971) psychobiological model of esthetic experience. Thus, following a comparative approach (Marin, 2015), we systematically examined the nature of hedonic tone in the experience of affective environmental scenes, environmental scenes converted to cartoons, representational paintings as well as music. One single factor of esthetic preference has previously been proposed, and beauty has been described as the concept best representing this factor (Eysenck, 1940; Marty et al., 2003; Jacobsen et al., 2004; Augustin et al., 2012a,b). Empirical evidence for this claim was usually based on results of a factor analysis, which showed that different evaluative scales loaded on one common underlying factor, and that beauty had the strongest loading on this factor. Consequently, researchers have tended to consider mainly beauty as a measure of hedonic tone in the study of esthetic experiences (Nadal et al., 2010). In the present study, we explored the possibility that hedonic tone has a multifaceted nature that cannot be ignored in modeling esthetic experiences.

Our primary hypothesis was that beauty and other measures of hedonic tone might not be similarly related to complexity if the latter is held constant. In a between-subjects design, subjective ratings of beauty, pleasantness and liking were collected for each stimulus set and correlated with ratings of complexity by controlling for familiarity effects. We predicted that the multifaceted nature of hedonic tone might be more evident in responses to paintings, cartoons and music than in environmental scenes depicting common, everyday life scenes. The different aspects of hedonic tone only became clearly apparent with regard to human-made visual artworks and music, for which we observed different relationships with complexity, ranging from positive and negative linear relationships, to quadratic relationships as well as indications of no relationship between these variables. Even subjective responses to cartoon-like pictures based on environmental scenes did not show a significant effect (although we observed weak indications), possibly due to the fact that cartoons were not considered as having a high esthetic quality or artistic status by participants.

With respect to Berlyne’s (1971) psychobiological model, our data suggests that the proposed inverted-U curve between complexity and hedonic tone may only be one out of several possible associations, depending on the measure of hedonic tone as well as the underlying dimension of complexity. However, the positive linear relationship between complexity and arousal was confirmed by our data across several stimulus categories and participants groups. As suggested by Nadal et al. (2010), sub-dimensions of visual complexity may show different associations with beauty ratings of visual stimuli. Here, we further demonstrate that the relationship with complexity (if held constant) may differ across measures of hedonic tone. Taken together, these results indicate that a differentiated view of concepts such as complexity and hedonic tone is warranted. It is plausible to assume that, for instance, dimensions of visual complexity (Nadal et al., 2010) show different relationships with beauty, liking and pleasantness. Likewise, the experience of musical complexity may by generated by several underlying dimensions whose associations with measures of hedonic tone may differ.

It is difficult to speculate why hedonic measures are not similarly associated with complexity, although they are themselves highly correlated in all experimental conditions and across stimulus sets. Esthetic experiences are currently being understood as interplay between emotion and cognition as well as interplay between bottom–up and top–down processes. For example, it is possible that beauty, pleasantness and liking differ in their affective connotations, with pleasantness presumably most closely linked to emotion. It can be surmised that the relationship between complexity and cognitive processing is partly determined by the affective content of the hedonic tone measures. Another related explanation refers to the different types of associations we observed between arousal and measures of hedonic tone in both paintings and music. If Berlyne was right to assume that arousal has a key function in esthetic experiences, then the nature of the arousal-hedonic tone relationship may play a role in the relationship between complexity and hedonic tone. Furthermore, it remains to be seen whether measures of hedonic tone contribute to one type of subjective experience in the perception of visual arts and music, which could be labeled as hedonic tone, or whether these concepts contribute to distinct experiences that are somewhat correlated and part of an esthetic experience, which may comprise other aspects than hedonic tone. This approach would also lead to a better understanding of whether and how visually and musically induced esthetic experiences differ.

To the best of our knowledge, no neuroimaging study has compared the activation patterns related to different hedonic concepts and complexity for the same picture set, let alone examined functional connectivity patterns. Our current results thus allow for the formulation of concrete hypotheses for future neuroimaging studies. For instance, since we present evidence that hedonic tone has a multifaceted nature in the experience of visual art and music, the respective underlying neural substrates may slightly differ and perhaps be dissimilarly connected to other brain regions involved in esthetic experiences, particularly those involved in structural feature processing. To be specific, paradigms used by Jacobsen and Höfel (2003) and Jacobsen et al. (2006) who compared the neural correlates of beauty judgments with those of symmetry judgments, may be extended by incorporating other measures of hedonic tone as well as by considering the multidimensionality of complexity (Nadal et al., 2010). We speculate that brain activations related to pleasantness may very likely differ from those related to beauty because the former can be considered as a dimension of core affect (Russell, 2009). This is also reflected in our data because we found a stronger correlation between pleasantness and arousal than between beauty and arousal. Further support for this argument comes from related research in the music domain. Brattico et al. (2013) developed a neurobiological model of esthetic experiences in music and argue that core affect is processed in brain areas such as the amygdala, sensory cortices and the parahippocampal gyrus, whereas esthetic judgments of beauty may primarily activate the orbitofrontal cortex, the anterior cingulate and the premotor cortex. Their model further proposes that brain areas active in beauty judgments may be active during reports of liking occurring during later processing stages. Reports of liking are also associated with activations in the ventral striatum and the insula. In order to fully understand this co-activation of brain areas involved in the processing of beauty and liking a systematic study of the time course of these processes will be crucial.

The role of arousal during esthetic processing has largely been ignored in neuroaesthetics, not only in the musical domain, as pointed out by Brattico et al. (2013), but also in the study of visual esthetic experiences (Leder et al., 2015), in which much emphasis has been placed on different aspects of valence, such as “positive vs. negative,” “happy vs. sad” or “pleasant vs. unpleasant.” In other words, a systematic study of the contributing affect systems during esthetic experiences that considers not only valence but also arousal is warranted (Russell, 1980). It may be true that in the context of Berlyne’s theory, which has arousal at its core, the relationship between hedonic tone and complexity may not always follow an inverted-U curve; however, arousal may still play a decisive role during esthetic episodes (Marin and Leder, 2013). Consequently, we highlight not only the need to study autonomic arousal but also the investigation of brain correlates of subjective arousal levels that may be associated with different neural affect systems (Koelsch et al., 2015) during esthetic experiences.

The present study also sheds more light on the role of familiarity in esthetic experiences and clearly indicates that it is necessary to account for familiarity effects even though participants may generally be unfamiliar with the stimuli, and as it was the case here, were non-experts in visual arts and non-musicians. In both the visual and musical domains, we observed on average moderate positive correlations between familiarity and measures of hedonic tone, and small negative correlations between familiarity and arousal as well as familiarity and complexity. Consequently, it is crucial to account for this effect by statistical techniques if one is interested in the nature of the complexity-hedonic tone relationship across stimulus categories and different participant groups.

We are aware of several limitations of the current study impeding broader generalizations. In both experiments, as well as in our previous work (Marin and Leder, 2013, 2016), we followed an affective approach by selecting stimuli varying in their affective contents (i.e., in arousal and pleasantness). It cannot be ruled out that our results are specific to these types of stimuli, and that findings for neutral stimuli may differ in the visual domain. Future studies may also involve male subjects and those showing high art interest or musical sophistication, other stimuli types, such as abstract artworks, and other measures of hedonic value, such as reward value. Furthermore, psychophysiological and neurophysiological measures may be added to the research design, especially since Berlyne (1974a) already proposed different psychophysiologcial signatures of measures of hedonic tone.

To conclude, this systematic study demonstrates that discrepancies regarding Berlyne’s psychobiological model might partly be rooted in the largely ignored multifaceted nature of hedonic tone. Future research, especially in the growing field of neuroaesthetics, may thus explore these nuanced aspects in greater depth.

## Author Contributions

MM designed the experiment and created the stimulus library. AL, MW, and MM collected the data. MM analyzed and interpreted the data. MM drafted the work and revised it after feedback from AL, MW, and HL. All authors provided final approval and agree to be accountable for all aspects of the work.

## Conflict of Interest Statement

The authors declare that the research was conducted in the absence of any commercial or financial relationships that could be construed as a potential conflict of interest.
