# Supplementary material for: Berlyne Revisited: Evidence for the Multifaceted Nature of Hedonic Tone in the Appreciation of Paintings and Music
Source: Front Hum Neurosci. 2016 Nov 4;10:536. doi: 10.3389/fnhum.2016.00536 (PMC5095118; doi:10.3389/fnhum.2016.00536)
Supplement: Supplementary file 1 [file Table_1.docx]

*Supplementary Material*

Berlyne Revisited: Evidence for the Multifaceted Nature of Hedonic Tone in the Appreciation of Paintings and Music

Manuela M. Marin*, Allegra Lampatz, Helmut Leder

*Correspondence: manuela.marin@univie.ac.at, manuela.marin@uibk.ac.at

1 Supplementary Table. Art interest questionnaire (in German).

*Teil 1: Bitte kreuzen Sie an, wie sehr die folgenden Aussagen auf Sie zutreffen.*

|  | Stimmt | | | | | | |
| --- | --- | --- | --- | --- | --- | --- | --- |
|  | Überhaupt  nicht völlig | | | | | | |
| 1. Um mir zu gefallen, muss ein Kunstwerk in erster Linie schön sein. | o | o | o | o | o | o | o |
| 2. Ich habe den Kunstunterricht in der Schule genossen. | o | o | o | o | o | o | o |
| 3. Ich unterhalte mich gerne mit anderen Leuten über Kunst. | o | o | o | o | o | o | o |
| 4. Ich habe viele Freunde/Bekannte, die sich für Kunst interessieren. | o | o | o | o | o | o | o |
| 5. Hässliche Kunstwerke kann ich nicht leiden. | o | o | o | o | o | o | o |
| 6. In der Kunst sollte es um eine genaue Darstellung der Umwelt gehen. | o | o | o | o | o | o | o |
| 7. Ich interessiere mich für Kunst. | o | o | o | o | o | o | o |
| 8. Kunst sollte in erster Linie dekorativ sein. | o | o | o | o | o | o | o |
| 9. Ich suche immer wieder neue künstlerische Eindrücke und Erlebnisbereiche. | o | o | o | o | o | o | o |
| 10. Im Alltag fallen mir spontan Kunstobjekte, die mich interessieren, auf. | o | o | o | o | o | o | o |
| 11. Ich komme aus einer kunstinteressierten Familie. | o | o | o | o | o | o | o |

*Teil 2: Bitte kreuzen Sie bei den folgenden vier Fragen die für Sie am besten passende Antwortmöglichkeit an.*

12. Wie oft besuchen Sie durchschnittlich Kunstmuseen bzw. Kunstgalerien?

| Seltener als einmal pro Jahr | Einmal pro Jahr | Einmal im Halbjahr | Einmal in 3 Monaten | Einmal im Monat | Einmal in 2 Wochen | Einmal pro Woche oder öfter |
| --- | --- | --- | --- | --- | --- | --- |
|  |  |  |  |  |  |  |

13. Wie oft lesen Sie Bücher, Zeitschriften oder Kataloge über Kunst?

| Seltener als einmal pro Jahr | Einmal pro Jahr | Einmal im Halbjahr | Einmal in 3 Monaten | Einmal im Monat | Einmal in 2 Wochen | Einmal pro Woche oder öfter |
| --- | --- | --- | --- | --- | --- | --- |
|  |  |  |  |  |  |  |

14. Wie oft sehen Sie sich Abbildungen von Kunstwerken an (Bildbände, Internet, etc.)?

| Seltener als einmal pro Jahr | Einmal pro Jahr | Einmal im Halbjahr | Einmal in 3 Monaten | Einmal im Monat | Einmal in 2 Wochen | Einmal pro Woche oder öfter |
| --- | --- | --- | --- | --- | --- | --- |
|  |  |  |  |  |  |  |

15. Wie oft besuchen Sie Veranstaltungen zu Kunst oder Kunstgeschichte?

| Seltener als einmal pro Jahr | Einmal pro Jahr | Einmal im Halbjahr | Einmal in 3 Monaten | Einmal im Monat | Einmal in 2 Wochen | Einmal pro Woche oder öfter |
| --- | --- | --- | --- | --- | --- | --- |
|  |  |  |  |  |  |  |

*Die folgenden Items müssen umgekehrt kodiert werden: 1, 5, 6 und 8.*
